# Bio-ethanol production by a novel autochthonous thermo-tolerant yeast isolated from wastewater

**DOI:** 10.1186/2052-336X-12-107

**Published:** 2014-09-25

**Authors:** Azadeh Tofighi, Mahnaz Mazaheri Assadi, Mohammad Hosein Arash Asadirad, Shohreh Zare Karizi

**Affiliations:** Department of Biology, Islamic Azad University, Varamin-Pishva Branch, Pishva, Iran; Departments of Biotechnology, Iranian Research Organization for Science and Technology, Tehran, Iran; Departments of Microbiology, Islamic Azad University, Zanjan Branch, Zanjan, Iran

**Keywords:** Ethanol, Isolation, Stress, Thermo stability, Wastewater, Yeast

## Abstract

**Background:**

It has been known for years that ethanol is a bio-fuel to replace fossil fuels**.** The ethanol industry requires the utilization of micro-organisms capable production with stresses. The purpose of present study was to isolate and characterize ethanologenic yeast with high potential application at high temperature to produce bio-ethanol.

**Methods:**

To isolate ethanologenic yeasts, wastewater samples from a starch producer plant in Varamin, Iran were used. The isolates were identified by molecular characterization. Characteristics of the isolated strains were determined at 30, 35, 40 and 45°C for 48 hours.

**Results:**

50 yeast strains capable of growing well in agar plates in a temperature range of 30–45°C were isolated. Out of the isolated strains, only three strains were screened for their ability to grow at 45°C. Selected yeast, designated as AT-3 strain which showed efficient flocculation capabilities with higher ethanol production and grew faster as compared to the rest of strains in media with 180 g/L glucose at 35°C. The selected yeast was identified as a new strain of *Saccharomyces cerevisiae* and submitted to the Gene-Bank database. Its’ optimum growth temperature was between 35 and 40°C. The results showed that during the bio-ethanol production 2.5 × 10^10^ and 8.5 × 10^9^ (CFU/mL) were a good indication of strain capability in heat tolerance. Also, ethanol produced at a raise of 6.9% and 6.85% (w/v) at 35 and 40°C, respectively, whereas glucose-to-ethanol conversion yield was about 75% of the theoretical value.

**Conclusions:**

Results emphasized that the isolated strain identified as *Saccharomyces cerevisiae*. This specific strain has thermo-tolerant, osmo-tolerant, flocculating capabilities with potential for application in developing a low cost ethanol industry.

## Background

Ethanol is an important liquid product with potential as a bio-fuel to replace fossil fuels [1]. To be able to substitute bio-ethanol for gasoline, one must overcome many obstacles including low cost bio-ethanol production [2].The ability of microbes for adaptation can be used for decreasing ethanol prices [3]. Industrial ethanol production depends on microbial activity, particularly that of yeasts. In industrial ethanol production, there are many important factors which should be considered. Temperature is one of the most important environmental factors affecting microbial activity [4]. Two of the problems associated with fermentation of sugar are the high temperatures (35-45°C) and high ethanol concentration (over 20%) [5]. Like all microorganisms, yeasts exhibit specific characteristics when growing at different growth temperatures [6]. Thermo-tolerant yeast refers to the yeasts possess optimum temperatures values above 40°C, as well as, the maximum temperature values range from 35°C up to 40–41°C for *Saccharomyces cerevisiae* strains [7, 8].Using thermo-tolerant yeasts for bio-ethanol production have several benefits: (i) Rapid metabolic activity, high fermentation rate and output. (ii) Reduction of gas solubility. (iii) Decreasing the viscosity of the media along with increasing temperature. (iv) Reduction of energy requirement. (v) Minimizing the chance of contamination [2, 3, 5, 6, 9].

The ability of micro-organisms to adapt to different temperature environments has attracted considerable attention. Ethanol production in the world is sustained by fermentation with ethanologenic yeast. The growth of yeasts varies according to temperature [4, 8]. In many countries, summer temperatures frequently reach over 35°C. Cooling costs during the process of ethanol production are expensive. Therefore, thermo-tolerant ethanologenic yeast strains would be useful for reducing the production cost [2, 3]. Tolerance to high temperatures and ethanol concentrations are important factors of microorganisms for increasing efficiency on the industrial scale [1, 5].

Therefore using the micro-organism with tolerance toward the inhibitors like high temperature, can increase the yield of ethanol production [5, 10] and decrease the price of production [2]. The aim of this study was to isolate and screen indigenous thermo-tolerant yeasts producing ethanol at high temperature from wastewater in the city of Varamin in Iran during summer season.

## Materials and methods

### Media

The media and chemicals were purchased from Merck (Germany). The main sources of all the chemicals concentrations for culture medium were chosen based on the literature [11]. Rose Bengal Chloramphenicol Agar was used for yeast isolation, which consisted of 10 g glucose; 5 g papaic digest of soybean meal, 1 g KH_2_PO_4_, 0.5 g MgSO_4_, 7 H_2_O and 15 g agar in 1 litre distilled water. 0.05 g/L Rose Bengal and 0.5 g/L Chloramphenicol were also added for the inhibition of fast growing fungi and bacterial growth [11]. Potato Dextrose Agar (PDA) medium containing 300 g potato, 20 g Dextrose and 20 g agar in 1 litre distilled water was used for preparing inoculums. Two other media were used for screening process. These two media were designated as pre-culture and fermentation media. Pre-culture medium, consisted of 30 g glucose, 10 g yeast extract, 0.6 g ammonium phosphate and 1.2 g ammonium sulphate, per litre. A synthetic medium used for fermentation which consisted, per litre, 180 g glucose, 10 g yeast extract, 0.6 g ammonium phosphate and 1.2 g ammonium sulphate. These screening media were adjusted to pH 5.5 with 1 N HCl. All the media were autoclaved at 121°C and 15 Lb pressure for 15 minutes. For each purpose, experiments were performed in triplicates. The medium was prepared as reported by Tofighi et al. [11].

### Sampling

To isolate ethanol producing yeasts, wastewater samples (200 mL) were collected from ten different sites of same sampling station in a starch producing plant in Varamin (Iran) on Jul. 2010, in to sterilized Erlenmeyer flasks, which were loosely covered and transported on ice to the laboratory within 1 h of collection. At the time of sampling some physicochemical parameters of effluents such as, temperature (°C), pH, BOD and COD were checked [12].

### Yeast isolation and maintenance

For balancing the buffering capacity, 90 mL phosphate buffer (pH: 7.0) was added to the samples and shaken vigorously for 1 h. After that, 100 μL of the supernatants were spread on sterilized Rose Bengal Chloramphenicol Agar plates. The cultivated media were incubated aerobically at 30, 35, 40 and 45°C for 3 days. Representative colonies were selected randomly on the basis of colony color and distinct morphological appearance, purified and observed under microscope. In this order, the classical methods described by Barnnet et al. were used [13]. Subsequently, they were stored in 20% glycerol at -75°C for further analysis [14] and deposited at PTCC (Persian Type Culture Collection).

### Molecular identification procedures

Total genomic DNA from the isolates was extracted [15]. Primarily, the selected yeast isolates were identified based on 18S rRNA gene (partial), ITS1, 5.8S rRNA gene, ITS2 and 26S rRNA (partial) gene. 18S rRNA gene was amplified by primers NS-1 (5′-GTAGTCATATGCTTGTCTC) as forward and NS-8 (5'-TCCGCAGGTTCACCTACGGA) as reverse primer. It was sequenced using D1/D2 domain of the 26S rRNA gene sequencing. 26S rRNA gene was amplified with NL-1 (5′-GCATATCAATAAGCGGAGGAAAAG) as a forward primer and NL-4 (5′-GGTCCGTGTTTCAAGACGG) as a reverse primer to specific differentiation ITS region of *S. cerevisiae*[16]. PCR was performed in a final volume of 50 μL containing 1× Buffer, 2.5 mM MgCl2, 250 μM (each) dNTP Mix, 1U Taq Polymerase, 0.2 μM of each primers and 20 ng of the extracted DNA. Amplifications were performed for 36 PCR cycles with denaturing at 94°C for 1 min, annealing at 52°C for 1 min, and extension at 72°C for 2 min, with the final extension for 10 min [17]. Polymerase chain reaction products were separated by 1.5% (w/v) agarose gel electrophoresis in 0.5× TBE buffer with ethidium bromide (05 μg/mL) [15]. Purification and sequencing were performed by the Iranian Biological Resource Center (IBRC).Research for DNA similarity was performed with the National Centre of Biotechnology Information Gene-Bank.

### Screening the thermo-tolerant, osmo-tolerant and ethanologenic yeasts

To prepare inoculums, pure isolated strains were streaked on sterilized PDA plates and incubated at 30°C. After 48 h incubation time, one loop-full of cells was transferred to 250 mL sterilized conical flask containing 50 mL pre-culture medium. The pre-culture media were incubated on rotary shaker at 30°C, 150 rev/min for 20 h. Then, the ability of the selected strains to produce ethanol was determined by transferring 6.16 × 10^7^ CFU/mL cells from pre- culture to fermentation media. Since the yeast cells should grow in aerobic conditions for the first 8 hours, the flasks were placed on a shaker-incubator at 30°C, 150 rev/min. After this period, the cultures were aseptically transferred to a 100 mL sterilized Erlenmeyer flask, equipped with rubber stoppers and sterile syringe needles. Subsequently, to complete the fermentation process and screen the thermo-tolerant yeasts, the fermentation medium was incubated at 30, 35, 40 and 45°C for a further 40 hours under anaerobic conditions [10]. All experiments were performed in triplicates.

### Analytical method

The sugar consumption during cultivation were quantified using an enzymatic method (Kit Glucose (GOD – PAP), Pars Azmun, Iran). Samples were aseptically taken to determine the optical density at 600 nm [18]. Ethanol concentrations were determined using gas chromatography on 14A Shimadzu as described previously by Tofighi et al. [11]. The bio-ethanol concentration in each sample was determined using a standard curve of ethanol.

## Results and discussion

### Isolation of yeast strains

During industrial bio-ethanol production, microorganisms are exposed to numerous environmental stresses such as high temperature and high sugar concentrations. Cellular micro molecules are seriously damaged under stress conditions, which leading to inhibition of cell growth and fermentation. To avoid lethal damage, bio-ethanol industry requires the utilization of microorganisms capable of working with stresses. Stress-tolerant microorganisms are thought to naturally occur; primarily in contaminated area. Studies suggested that physicochemical parameters of contaminated area, such as Hydrogen ion concentration (pH), can contribute to survive and tolerance of microorganisms against stresses. General effect of pH on cells is related to the rates of enzymatic reactions [19]. The measurement of Biological Oxygen Demand (BOD) and Chemical Oxygen Demand (COD) of the wastewater samples exhibited the rate of effluent pollution [20]. In the present study, the pH values of wastewater samples was about 4 and revealed no significant differences at all locations. Also, BOD and COD measurements was about 33 mg/L and 179 mg/L, respectively; which, indicated the wastewater samples were belonged to weakly contaminant wastewater [20]. Survival of microorganisms in the unfavorable conditions depends on their tolerance mechanisms. So, in this research, efforts were made to isolate and screen thermo-tolerant yeasts from wastewater. 50 colonies were isolated from studied industrial effluents.

### Selection and identification of the thermo-tolerant and osmo-tolerant yeast strains

Out of the 50 yeast isolated colonies, three strains showed high growth rate at 40 and 45°C on the solid medium. The selected colonies were designated as AT-3, AT-7 and AT-16. Among the isolated, AT-3 was capable of producing floccules whereas AT-7 and AT-16 did not flocculate in fermentation media.

Molecular identification of the isolates according to the 5.8S-ITS rRNA sequence analysis showed that the AT-7 and AT-16 strains belonged to the species *Candida tropicalis* and the strain AT-3 belonged to the species *Saccharomyces cerevisiae* which exhibited 95% homology with *Saccharomyces cerevisiae* with accession number of *GU080045.1*. Then, to achieve a certain identification of the yeasts D1/D2 region of 26S rRNA was sequenced and compared with those available in the EMBL nucleotide sequence database. Gene analysis of AT-3 strain based on 26S rRNA (partial) showed 98% phylogenetic relationships among strain AT-3 and *Saccharomyces cerevisiae* with accession number of *GQ376089.1*. The species-specific partial D1/D2 domain and 5.8S ITS region sequences were submitted to the Gene-Bank database as *Saccharomyces cerevisiae* AT-1350 under accession number: KF725624.

**Figure 1:**
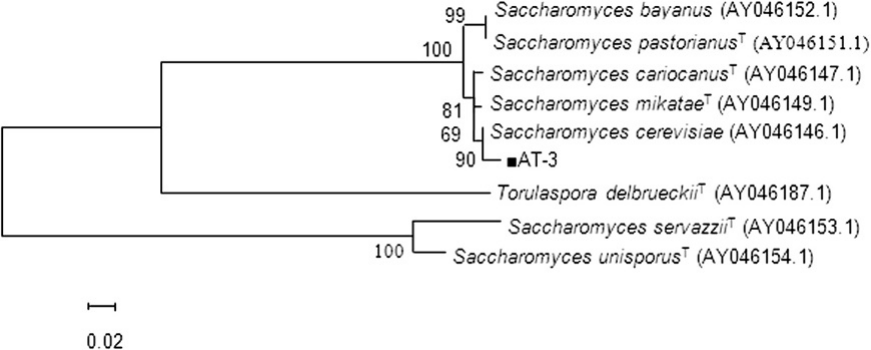
**Neighbour-joining tree of the AT-3 isolate and type strains of related genera.** Neighbour-joining tree based on 18S rRNA gene (partial), ITS1, 5.8S rRNA gene, ITS2 and 26S rRNA (partial) gene sequence showing the phylogenetic relationships among Strain AT-3 and type strains of related genera. Numbers at branch nodes are bootstrap values (percentages of 1000 replicates). Bar 2 substitutions per 100 nucleotide positions.

Phylogenetic tree of AT-3 strain was constructed using the neighbour-joining method and illustrated in Figure 1.

### Effect of high temperatures on growth

**Figure 2:**
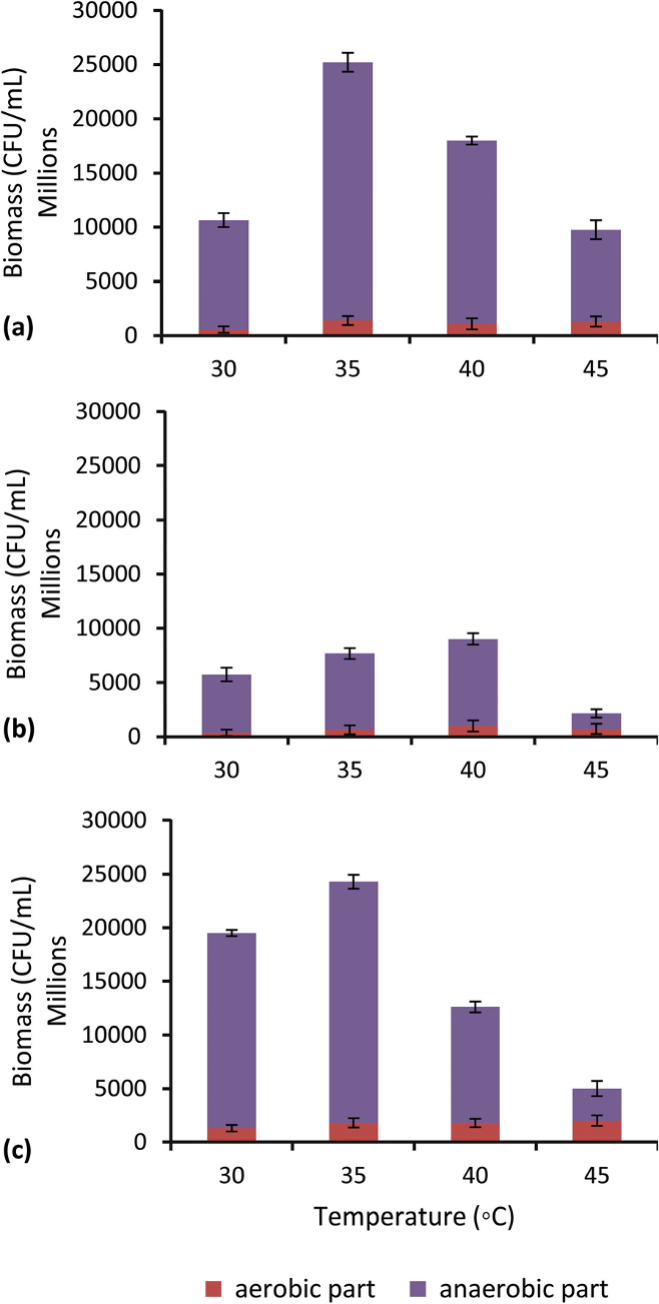
**Effect of temperature on growth of selected strains.** The growth of selected strains in different temperatures during the batch cultivation. Fermentation process was separated in aerobic (8 h) and anaerobic (40 h) parts. Error bars represent standard deviation of three replicates. **(a)**: AT-3; **(b)**: AT-7 and **(c)**: AT-17.

The cell mass of the yeast strains after 48 hours of incubation was measured by optical density at 600 nm. Moreover, at this time, pH values of the fermentation media was determined between 3.8-4.3. The influence of different temperatures (30, 35, 40 and 45°C) on growth of the isolated strains in the media with 180 g/L glucose, during the 48 h batch cultivation is displayed in Figure 2. As showed, the highest cell mass productivity was obtained at 30°C for AT-16 and at 35, 40 and 45°C for AT-3 strain. The results indicated that, the AT-3 strain is able to survive at higher temperatures. The highest cell mass productivity in anaerobic conditions was obtained at 35 and 40°C for AT-3. The cell mass in the first 8 hours (aerobic condition) and in the second part (40 h, anaerobic condition) was determined at the temperature studied. As presented in Figure 2, the cell mass productivity is affected with increasing the temperatures. These results agree with Ali Shah et al. [6] and Torija et al. [4] reported that yeast’s viability decreases and the fermentation process is inhibited as the temperature increases. This may be related to the passage of time (48 hours), reduction of pH [19], the differences between the regulatory elements of stress responses in aerobic and anaerobic conditions as well as their sensitivity to ethanol toxicity [21–23].

### Effect of high temperatures on ethanol productivity

**Figure 3:**
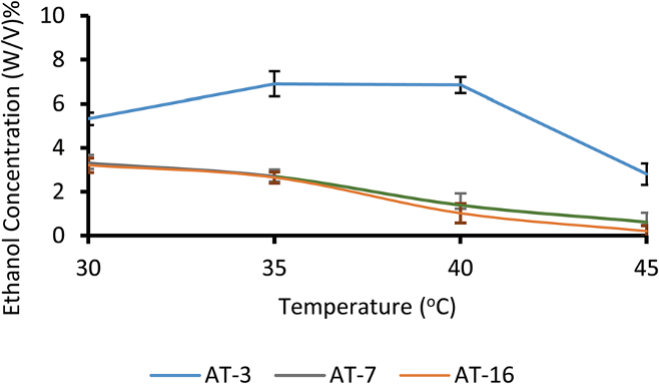
**Effect of temperature on ethanol productivity of the selected strains.** Ethanol concentrations of the selected strains in different temperatures during the batch cultivation after 48 hours. Error bars represent standard deviation of three replicates.

The effect of elevated temperature on ethanol productivity (w/v)% in isolated yeasts was investigated (Figure 3). As illustrated, the ethanol productivity within 48 hours was determined to be about 5.32, 3.3 and 3.2 (w/v)% for the AT-3, AT-7 and AT-16 strains at 30°C; 6.9, 2.69 and 2.65 (w/v)% at 35°C; 6.85, 1.4 and 1.02 (w/v)% at 40°C as well as 2.8, 0.62 and 0.2(w/v)% at 45°C, respectively. As shown, the AT-3 strain could also tolerate and produce ethanol at higher temperatures. Our results indicated that the optimum temperature of the AT-3 strain was between 30–40°C and showed a tolerance against high temperature. Also, the highest ethanol productivity within 48 hours was obtained for the AT-3 strain at 30°C. It rose up to 150% at 35°C and decreased sharply over this temperature. At the same time the AT-3 ethanol concentration rate (Figure 3) increased about 130% at 35°C and was maintained at 40°C, further decreased occurred slowly at 45°C. Yeast growth and ethanol production decreased at 45°C too. The cells remained active but not progressive at temperatures above that. Cimpeanu et al. [9] also isolated thermo-tolerant yeast belonged to *Saccharomyces cerevisiae*. They confirmed that, thermo-tolerant yeast could promote high yield of ethanol at high temperatures [9].

### Effect of high temperatures on glucose consumption

**Figure 4:**
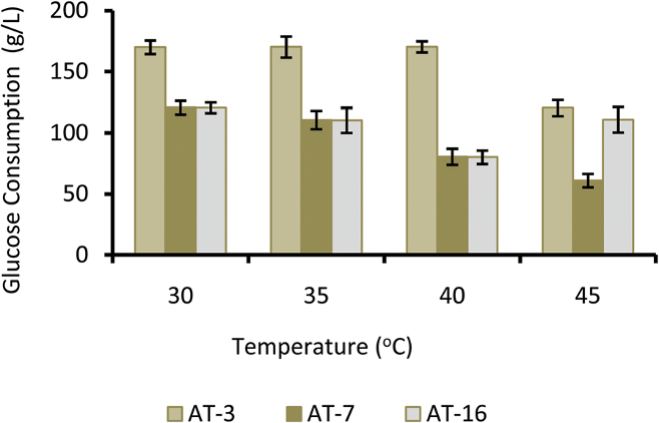
**Effect of temperature on glucose consumption of the selected strains in the fermentation media.** Glucose consumption of the selected strains in the media with 180 g/L glucose, in different temperatures during the batch cultivation after 48 hours. Error bars represent standard deviation of three replicates.

Media containing 180 g/L glucose was used in batch fermentations at the temperature range of 30–45°C for 48 h (Figure 4). As shown, glucose metabolism of AT-3 strain was stimulated from 17.14 -17.88 g/L, with increasing temperature from 30–35°C. When fermentation continued at temperatures over 35°C, production decreased rapidly. Glucose consumption of AT-7 and AT-16 strains was about 12.5 g/L at 30°C which decreased with increasing the temperature. In this work, we have shown that increasing the temperature up to 40°C did not have any negative effects on the AT-3 strain. Also, inhibition effects of the high glucose concentration were shown for the AT-7 and AT-16 strains. The highest metabolized glucose rate for AT-3 was obtained at 40°C and then at 35 and 30°C. This is may be because of the cells effort to survive at undesired conditions [24–26].

The data showed that the AT-3 strain could remain active at high temperatures to continue the fermentation process. Glucose-to-ethanol conversion yield of AT-3 was about 75% of the theoretical value at 35 and 40°C.

On the other hand, the AT-7 and AT-16 strains showed a good activity regarding the fermentation process using the media with high concentration of glucose at the range of 30–45°C.

The results indicated that the growth rate and ethanol productivity of the isolated yeasts were significantly affected by the temperature studied. The results showed that, in comparison with AT-7 and AT-16, AT-3 strain exhibited a good advantage at the highest temperature in the osmotic pressure, and we considered them osmo-tolerant and flocculating. These results were consistent with Kiran Sree et al. [10], and Nahvi et al. [27] studies which reported that flocculation ability could help the yeast strains to withstand the environmental pressures. The optimum temperature for the growth of AT-3 in aerobic conditions was between 35 and 40°C, for AT-7 and AT-16 was about 30°C.

The ability of microorganisms to adapt to different temperatures has attracted considerable attention, but the mechanism underlying this phenomenon is not well understood. Yeast cells exhibit a rapid molecular response when exposed to elevated temperatures [28]. Thermo-tolerance appears to involve a range of complex mechanisms [5, 23, 28, 29]. Several mechanisms have been reported to be associated with stress responses, based on the ability to produce flocculated cells [10, 27, 30] and/or cause changes in gene expression [31, 32], to affect membrane or cellular composition [23, 29], and induce heat shock proteins such as Hsp90 [33]. Markedly, the heat shock response in yeast is one of the best molecularly characterized responses of eukaryotic cells and has been widely reviewed [34]. Some researchers have demonstrated that in some yeasts, the heat shock elements (HSE) are unresponsive to other stress (osmotic, oxidative, DNA damage, glucose repression, etc.) [23]. Moreover, the stress of environmental changes and selective pressures can actually influence the evolutionary processes [33, 35]. Also, Kiran Sree et al. [10], and Cimpeanu et al. [9] indicated that, high concentration of glucose in the medium inhibited the growth of yeast cells and the production of ethanol, and the inhibitory effect is attributed to high osmotic pressure.

## Conclusions

In this study, the AT-3 strain was found as autochthonous yeast with tolerance against high glucose concentrations. It is a flocculent strain that belongs to *Saccharomyces cerevisiae* with the optimum temperature over 35°C. The growth rate and ethanol productivity are stable at high temperatures (between 35–45°C). The glucose-to-ethanol conversion yields are about 75% of the theoretical value at 35 and 40°C. According to the data, we would like to highlight the AT-3 strain as a novel, highly desirable thermo-tolerant, osmo-tolerant, and flocculent *Saccharomyces* for the low cost alcohol production industry especially in hot areas.
